# The impact of vaporization on adenoma weight in benign prostatic hyperplasia surgery

**DOI:** 10.1002/bco2.70171

**Published:** 2026-02-04

**Authors:** André B. Silva, Bruno R. Lebani, Eduardo R. Pinto, Luciano T. Silva, Denise S. Gouveia, Hudson de Lima, Marcia E. Girotti, Milton Skaff, Fernando G. Almeida

**Affiliations:** ^1^ Department of Urology Federal University of São Paulo São Paulo Brazil

**Keywords:** adenoma, benign prostatic hyperplasia, enucleation, transurethral resection of the prostate, vaporization

## Abstract

**Objective:**

The weight of adenoma removed during benign prostatic hyperplasia (BPH) surgery is commonly used as a surgical landmark. During endoscopic procedures, prostatic tissue is exposed to heat generated by electric current, resulting in tissue vaporization that may affect the final specimen weight. This study aimed to quantify tissue loss due to vaporization during TURP, comparing monopolar and bipolar techniques.

**Materials and Methods:**

Surgical specimens from 32 patients undergoing open simple prostatectomy were analysed. After enucleation, adenomas were weighed and then completely resected in vitro simulating TURP using monopolar or bipolar energy. The weight of the resected fragments was measured to estimate tissue loss associated with each energy source and compared with preoperative MRI‐estimated transition zone volumes.

**Results:**

Mean patient age was 68.2 ± 5.75 years. There was a strong correlation between MRI‐estimated transition zone weight and enucleated specimen weight (97.5 ± 40.1 g vs. 90.5 ± 38 g; *r* = 0.998, *p* < 0.001). Of the 32 enucleated adenomas, 16 were resected in vitro using monopolar energy and 16 using bipolar energy. After in vitro resection of the adenoma, a significant decrease in the enucleated specimen weight was observed (from 90.5 ± 38 g to 64.25 ± 25.6 g; *p* < 0.001). Overall, the mean decrease in weight after resection was 26.3 g, corresponding to a 29% reduction. Bipolar resection showed a greater reduction in tissue weight compared to monopolar resection, with a decrease of 36.8% and 19.4%, respectively (*p* < 0.001).

**Conclusion:**

The weight of the adenoma removed during surgical treatment of BPH differs depending on the technique used and, therefore, cannot be used comparatively between techniques. Resection surgeries result in tissue vaporization and dehydration, reducing adenoma weight by approximately 28%. Adenoma volume determination by MRI shows a strong correlation with the volume to be removed during surgery.

## 1 INTRODUCTION

Benign prostatic hyperplasia (BPH) is a common urological condition visually characterized by increased prostate volume (PV), which plays a critical role in surgical decision‐making according to major guidelines, guiding the choice of surgical technique.^1^, ^2^ The amount of tissue to be removed in prostate surgery can be estimated based on PV, more precisely by the volume of the transition zone (TZV). The impact of complete versus partial removal of the adenomatous tissue on functional outcomes remains a matter of debate in the literature.^3^, ^4^ However, the rationale remains the complete removal of the prostatic adenoma as a therapeutic target.

Among surgical techniques, simple prostatectomy achieves the greatest adenoma removal, typically excising the entire transitional zone, which corresponds approximately 85%–90% of the preoperatively estimated total prostate volume (TPV) in glands larger than 80 cc.^5^ Transurethral resection of the prostate (TURP), the gold standard for prostates smaller than 80 cc, removes approximately 50% of the TPV under optimal conditions.^6^ Endoscopic enucleation of the prostate (EEP) mimics simple prostatectomy and achieves an average removal of about 72% of the TPV.^7^, ^8^, ^9^ Resection and enucleation techniques are based on distinct surgical principles, with different anatomical landmarks, which clearly influence the extent of tissue excised.

Several studies have supported EEP as an alternative to TURP or simple prostatectomy.^9^ Despite ongoing debate in the literature regarding functional outcomes, EEP has been frequently mentioned as more effective than TURP based on removing a greater amount of prostatic tissue. It is well recognized that during endoscopic surgeries, the prostatic tissue is continuously exposed to heat generated by electric current, leading to vaporization of a portion of the tissue, which might impact the final weight of the removed specimen.^10^, ^11^

The objective of this study was to quantify the amount of tissue loss due to vaporization during TURP, comparing both monopolar and bipolar techniques. We aimed to estimate the actual amount of adenoma removed, enabling more realistic standardizations and comparisons between techniques.

## 2 METHODS

This is a prospective and experimental study conducted in a single centre (São Paulo, Brazil) from November 2022 to March 2024. Ethical guidelines were followed in accordance with Good Clinical Practice guidelines and the Declaration of Helsinki. Patients were selected after obtaining ethical approval by the Research Ethics Committee from Hospital São Paulo, with free and informed consent signed by all patients. The study was registered in the Clinical Trial Registry (UTN – U1111‐1288‐1500).

Patients with symptomatic benign prostatic hyperplasia refractory to medical therapy, with large prostate volumes (>80 g on MRI), and who were candidates for open simple prostatectomy (Freyer's method) were eligible for inclusion. Patients with prostate cancer and those who declined to participate in the study were excluded. All patients underwent magnetic resonance imaging (MRI) within 3 months prior to surgery for accurate estimation of TPV and TZV measurements using the ellipsoid formula.^12^ The transition zone index (TZI) was calculated according to the formula TZI (%) = TZV/TPV. In addition, all patients had a transabdominal ultrasound performed as part of the initial evaluation within 3 months prior to MRI, performed by a radiologist.

**FIGURE 1:**
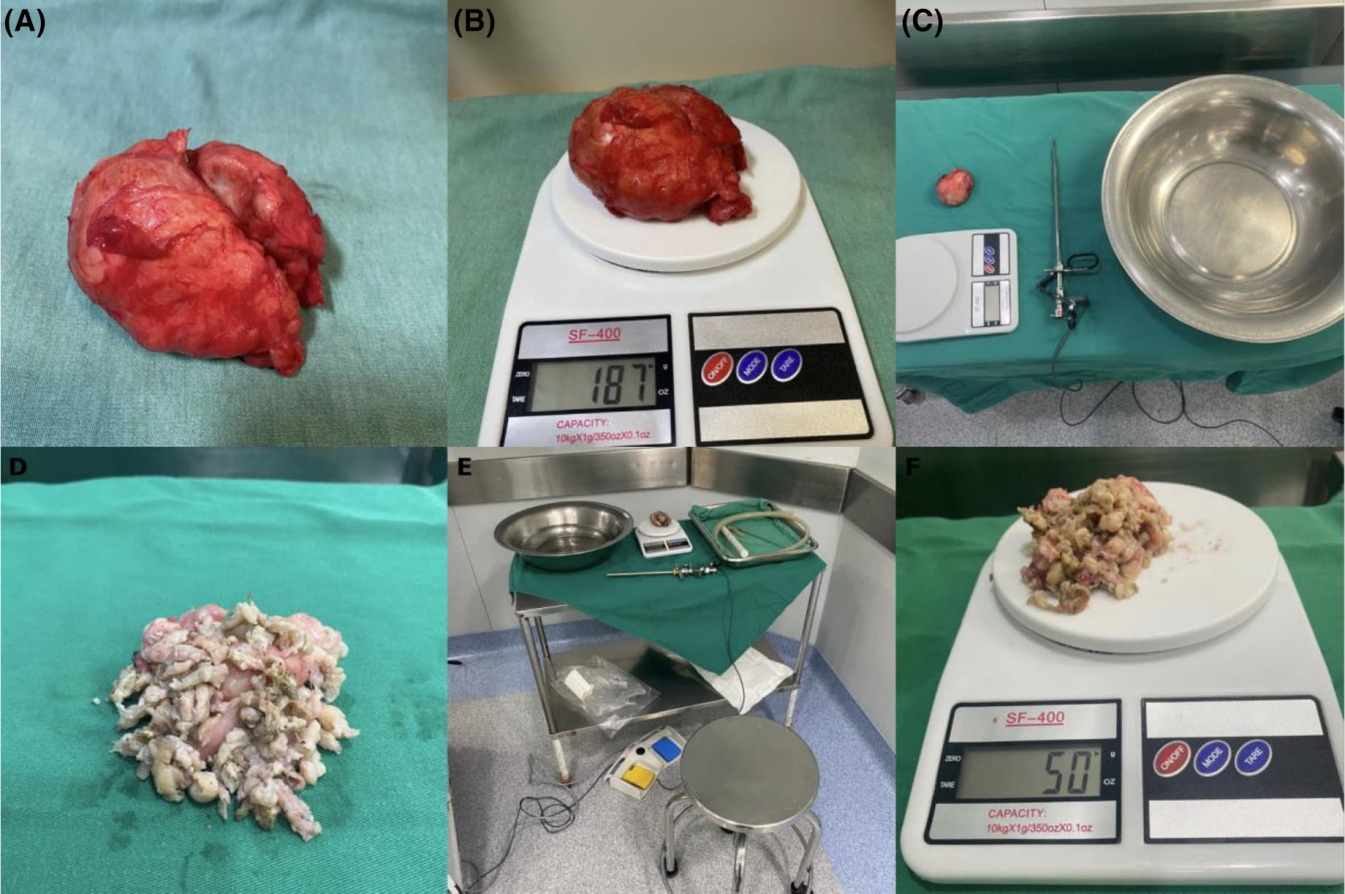
Step by step: bench setup. (A) Enucleated adenoma; (B) adenoma being weighed; (C) desktop setup; (D) fragments after in vitro resection; (E) bench setup; (F) fragments being weighed.

Through a midline suprapubic incision, a transvesical simple prostatectomy was performed. Immediately after the procedure, the enucleated adenoma was wrapped in a dry surgical compress to remove excess surface fluid and weighed on a precise scale (0.1 g). Subsequently, in a separate operating room, the adenoma specimen underwent in vitro resection simulating TURP, with half of the cases performed using monopolar energy and the remaining half using bipolar energy devices (Figure 1). All in vitro resections were performed by the same surgeon, a urologist from the department. The monopolar resection was performed with the Covidien Force FX (Electrosurgical Generator), set at 80 W cutting power and zero coagulation. The bipolar resection was performed with the Karl Storz Autocon® 400 (UH400U), also set at 80 W cutting power and zero coagulation.

The primary outcome was the percentage of prostatic tissue vaporized after resection, defined as the proportion of adenoma tissue removed during in vitro resection relative to the total weight of the enucleated adenoma. The secondary analysis was the comparison of vaporized tissue percentage between monopolar and bipolar resection techniques.

In vitro monopolar resection was performed in a stainless steel bowl filled with 3 L of 1.5% glycine solution at room temperature, simulating the fluid environment used in practical settings. An electrocautery grounding plate was connected to the bowl. Resection loops previously used in institutional procedures were reused. The adenoma was stabilized and maintained in contact with the surface using a manually controlled mobile rod, and was completely resected, generating tissue fragments similar in size to those typically produced during in vivo TURP. In vitro bipolar resection was performed using 0.9% normal saline solution, without the need for a grounding plate to complete the circuit. After complete resection, the tissue fragments were removed from the bowl using a sieve, wrapped in a dry compress to remove excess fluid, weighed and the values recorded. Subsequently, the fragments were preserved in 10% buffered formalin and sent to the pathology laboratory for routine histopathological analysis.

Sample size calculation was performed using G*Power software (version 3.1), based on the expected difference in tissue loss between monopolar and bipolar energies. A minimum absolute difference of 10%, with an assumed standard deviation of 10%, was adopted according to prior estimates.^10^, ^11^ With a statistical power of 95% and a two‐sided alpha level of 0.05, a minimum sample size of 13 patients per group was required. To account for potential losses and exclusions, the final sample size was increased by 20%.

Statistical analysis was performed using SPSS (V.9, SPSS Inc., Chicago, Illinois, USA) and Jamovi software (Version 2.3.28.0). The Shapiro–Wilk test and histogram analysis were applied to assess the normal distribution of variables. Descriptive analyses were performed using Student's *t*‐test or Mann–Whitney test for numerical variables and chi‐square to compare categorical variables. Spearman's correlation coefficient and linear regression were used to analyse the relationship between continuous numerical variables. Statistical significance was defined at *p* ≤ 0.05.

## 3 RESULTS

Between November 2022 and March 2024, a total of 34 patients were included in the study and underwent open simple prostatectomy followed by immediate in vitro resection. Two patients were excluded following histopathological analysis confirming prostate adenocarcinoma. As a result, 32 patients were included in the final analysis. The first 16 patients consecutively underwent resection using a bipolar device, while the subsequent 16 were treated using a monopolar device.

The mean age ± standard deviation (range) of the patients was 68 ± 5.75 (58–80) years. The mean total prostate volume (TPV) estimated on preoperative MRI was 129 ± 45 cc. The median IPSS was 15 (0–31), and the median PSA level was 6.67 ng/dL (2.3–29.5). The demographic and clinical characteristics of the total sample, as well as those stratified by surgical technique (monopolar or bipolar), are presented in Table 1.

**TABLE 1:** Characteristics of clinical variables.

(*n*)	Total (32)	Monopolar (16)	Bipolar (16)	*p*
Age, years	68 ± 5.75	67.8 ± 5.77	68.2 ± 5.91	0.834
BMI (kg/m^2^)	27 ± 3.44	27.6 ± 4.09	26.5 ± 2.67	0.398
IPSS	15 (0–31)	15 (0–28)	17 (0–31)	0.611
PSA (ng/dL)	6.67 (2.3–29.5)	7.31 (2.8–29.5)	5.71 (2.3–12)	0.149
TPV US (cc)	131 (52–271)	133 (52–271)	114 (89–193)	0.22
TPV MRI (cc)	129 ± 45	115 ± 28	142 ± 54.9	0.181
TZV MRI (cc)	97.5 ± 40.1	83.8 ± 28.5	111 ± 45.9	0.052
TZI (%)	74.3 ± 9.81	71.2 ± 8.09	77.4 ± 10.6	0.076
IPP (cm)	1.3 ± 1.1	1.3 ± 1.3	1.3 ± 1	0.927

Abbreviations: BMI = body mass index, kg/m^2^; IPP = intravesical prostatic protrusion; IPSS = international prostate symptom score; IUC = indwelling urinary catheter; MRI = magnetic resonance imaging; PSA = prostate specific antigen; TPV = total prostate volume; TURP = transurethral resection of the prostate; TZI = transition zone index; TZV = transition zone volume; US = ultrasound.

**FIGURE 2:**
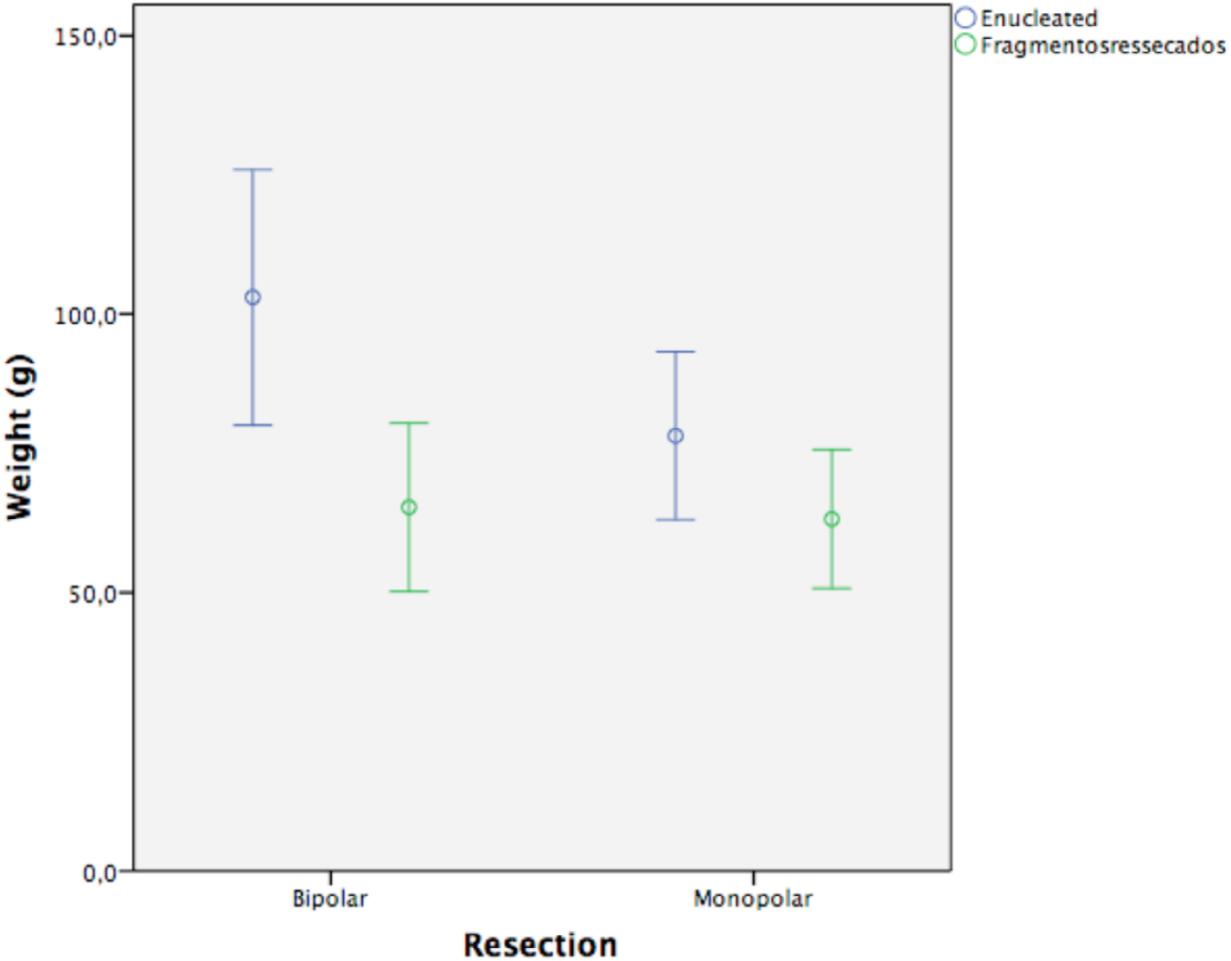
Weight before and after resection according to technique.

The mean adenoma weight before and after resection was 90.5 ± 38 g and 64.25 ± 25.6 g, respectively (Table 2), corresponding to a mean reduction of 29.05% and an absolute difference of –26.31 g (95% CI: 20.55–32.06; *p* < 0.001). On average, the resected adenoma accounted for 72 ± 9.17% of the enucleated adenoma. Stratified by technique, monopolar resection preserved 80.7 ± 1.8% of the original weight, whereas bipolar resection preserved 63.2 ± 2.7%, yielding a significant mean difference of 17.5% (*p* < 0.001; Figure 2). This corresponded to a 19.3% weight reduction with monopolar energy and 36.8% with bipolar.

**TABLE 2:** Mean and standard deviation of enucleated and resected adenoma.

	Total (*n* = 32)	Monopolar (*n* = 16)	Bipolar (*n* = 16)	*p*
Weight of enucleated prostate (g)	90.5 ± 38	78.1 ± 28.3	103 ± 43	0.063
Weight of resected prostate (g)	64.2 ± 25.6	63.2 ± 23.4	65.3 ± 28.4	0.819
Resected/enucleated (%)	72 ± 9.1	80.7 ± 1.7	63.2 ± 2.7	<0.001
Enucleated/TZV MRI (%)	92.6 ± 2.1	92.7 ± 2.13	92.5 ± 2.12	0.78

Abbreviations: MRI = magnetic resonance imaging; TZV = transition zone volume.

The mean adenoma weight estimated by MRI was 97.5 ± 40.1 g, whereas the actual weight of the adenoma following simple prostatectomy was 90.5 ± 38 g, representing a percentage difference of 7.1% and a mean absolute difference of 6.93 g (95% CI: 5.88–7.98; *p* < 0.001), with excellent correlation between the two methods (*r* = 0.999; *p* < 0.001; Table 3). Prostate weights estimated by ultrasound and MRI showed a strong correlation (*r* = 0.692; *p* < 0.001), with a mean difference of 1.81 g (*p* = 0.769).

**TABLE 3:** Prostate measurements.

	Mean	SD	% difference	Mean difference	95% CI		*p*	*r*	*p*
					Inferior	Superior			
TPV US	130.7	43.2	1.4	−1.8	−10.6	14.3	0.769	0.692	<0.001
TPV MRI	138.9	45.1							
TVZ MRI	97.5	40.1	7.1	−6.9	5.8	7.9	<0.001	0.999	<0.001
WEP	90.56	37.9							
WEP	90.5	37.9	29.1	−26.3	20.5	32.1			
WRP	64.2	25.6							
TPV MRI	128.9	45.1	24.3	−31.4	27.3	35.4			
TZV MRI	97.5	40.1							
TPV MRI	128.5	45.1	29.8	−38.3	33.9	42.6			
WEP	90.5	37.9							
TPV MRI	128.9	45.1	50.1	−64.6	56.1	73.2			
WRP	64.2	25.6							

Abbreviations: MRI = magnetic resonance image; TPV = total prostate volume; TVZ = transition zone volume; US = ultrasound; WEP = weight of enucleated prostate; WRP = weight of resected prostate.

**FIGURE 3:**
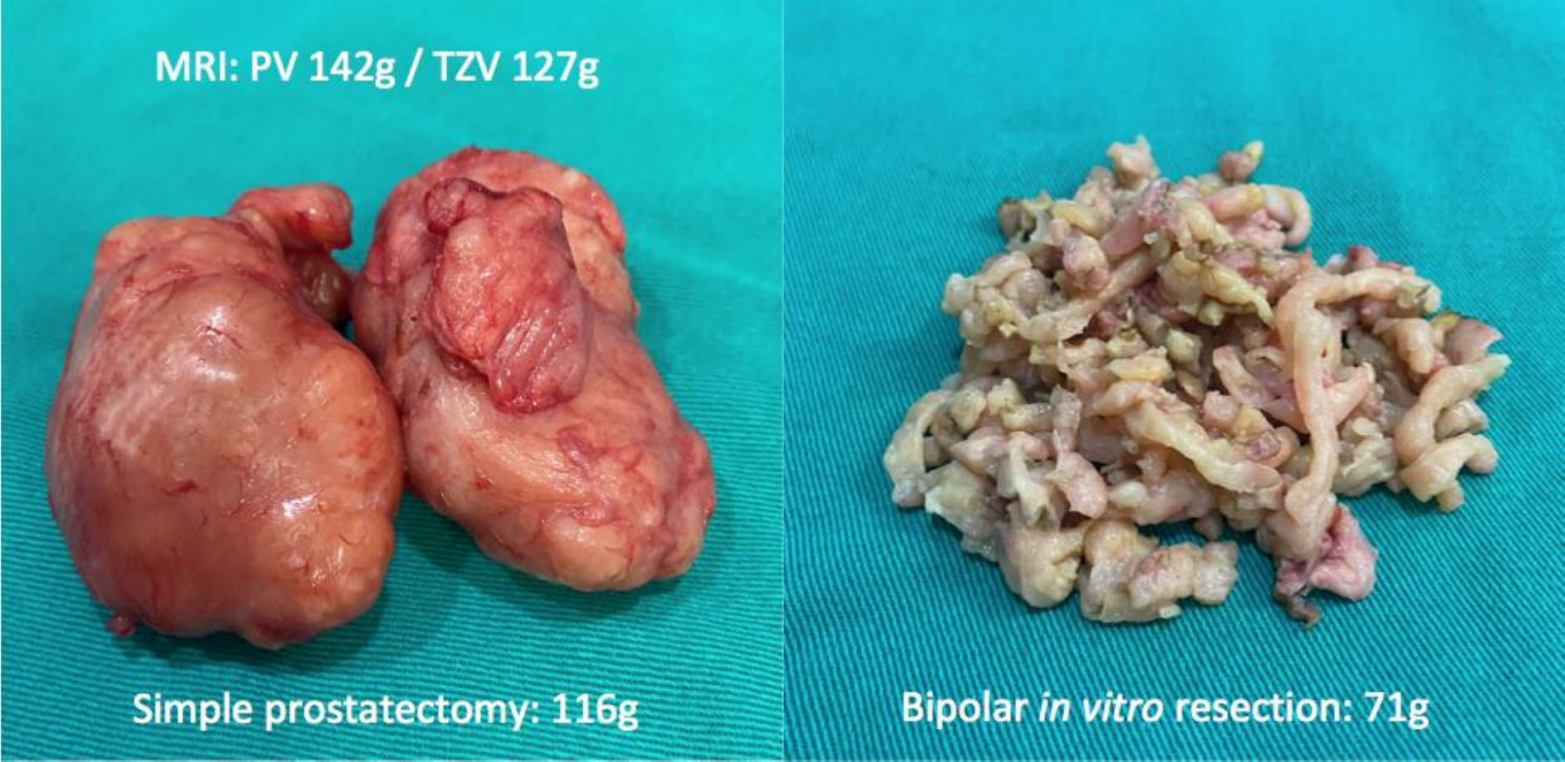
Photographic documentation of the enucleated adenoma that was subsequently resected in vitro with the corresponding measurements from preoperative MRI.

Figure 3 illustrates one of the study cases, showing a photographic record of the enucleated adenoma and the tissue fragments obtained from the subsequent bipolar in vitro resection, along with the respective weights and the preoperative MRI‐derived measurements.

## 4 DISCUSSION

To the best of our knowledge, this is the first study to assess the difference between enucleated prostate weight following simple prostatectomy and the sum of fragment weights after in vitro resection of the same adenoma, using MRI as a reference, to determine the percentage of tissue vaporized during endoscopic resections. The practical goal of this study is to provide surgeons with a better understanding of TURP specimens' weight when comparing to imaging measurements and enucleation techniques.

Several key aspects strengthen the study's findings. First, the comparison between monopolar and bipolar energy sources is clinically relevant, given their distinct electrosurgical mechanisms and tissue effects, and because much of the available literature is based on outdated monopolar technology. The in vitro resection model was designed to approximate clinical TURP conditions by using freshly enucleated adenoma tissue, standard immersion fluids, and energy settings commonly applied in routine practice. Nevertheless, the ex vivo setting does not fully capture variables inherent to the surgical act, including tissue vascularization, active bleeding, and the dynamic interaction between irrigation fluids and viable tissue, all of which may influence vaporization and coagulation behaviour in vivo. Despite these limitations, there is currently no feasible methodological approach that allows direct, complete, and accurate quantification of tissue vaporization under true in vivo conditions.

We found a significant difference between the prostate specimen weight from simple prostatectomy and the sum of in vitro resected fragments, with an average reduction of 28 ± 9.17%. This reduction was greater with bipolar energy (36.8 ± 2.72%) than with monopolar (19.3 ± 1.78%, *p* < 0.001), confirming substantial tissue loss due to vaporization and dehydration. The only study that analysed the vaporization potential with bipolar energy was the study by Khorrami et al.,^13^ which recorded a reduction of 21.3 ± 3.7% and 25.3 ± 2.5% in prostate weight when using monopolar and bipolar energies, respectively. These results support earlier estimates from the 1950s to 1960s indicating a 20%–33% reduction during experimental resections.^10^, ^11^ Historical studies on this topic were conducted under markedly different conditions. In 1950, Rubin described a single in vitro monopolar resection without fluid immersion.^10^ Whisenand et al. (1960s) resected cadaveric prostates using monopolar energy but lacked statistical analysis.^11^ Other 1970s studies used monopolar resection on freshly excised glands. Tissue weight reduction is attributed to fluid loss from prostatic acini and heat‐induced vaporization. In 1971, Lewis Hahn et al. found a 21% weight loss after resection and 15% after cold‐blade cutting, suggesting acinar destruction.^13^, ^14^ Fluid opalescence during resection supported this theory.

Tissue vaporization remains understudied. In 2012, Fagerström et al. evaluated muscle and kidney tissues resected in vitro using monopolar and bipolar energy.^15^ They found that 52% of muscle and 32% of renal tissue mass was vaporized, with greater vaporization using bipolar energy, independent of irrigation solution, highlighting the impact of tissue composition and energy type. Bipolar energy uses high‐frequency current confined between two nearby poles, allowing precise heat application and efficient vaporization. Saline solutions used with bipolar resection are excellent conductors, unlike the non‐conductive fluids required for monopolar resection, which reduce energy transfer efficiency.^16^, ^17^, ^18^

These findings raise important questions about a key surgical outcome: the amount of tissue removed. Many studies fail to standardize this measurement, typically reporting absolute weight without considering percentage of total or transition zone volumes.^7^, ^8^, ^9^ The concept of variable transition zone index (TZI) based on prostate size is underrecognized, despite its relevance to determining surgical targets. Additionally, tissue vaporization is often ignored when comparing techniques.

Simple prostatectomy is the gold standard for complete transition zone removal, accounting for 85% of total prostate volume (TPV).^5^ Endoscopic enucleation techniques such as HoLEP and BipoLEP achieve 70% TPV removal.^7^, ^8^, ^9^ Fong et al. concluded that at least 0.56 times of the TPV should be removed during EEP.^19^ TURP estimates vary widely: One large study found that no patient had more than 50% TPV removed, regardless of apparent completeness.^6^ The best estimates suggest TURP removes approximately 64% of TPV, with no clear difference between monopolar and bipolar methods.^20^ None of the BPH studies, when making comparisons between different methods, consider possible measurement biases.

Assessment of TURP completeness remains heterogeneous, as multiple approaches have been employed, including gross specimen weight, percentage of tissue removal relative to total prostate volume, intraoperative endoscopic impression, and postoperative imaging. While the intraoperative endoscopic appearance traditionally guides the surgeon in defining resection adequacy, postoperative imaging—particularly transrectal ultrasound or magnetic resonance imaging—has been increasingly used to estimate residual transition zone volume and objectively quantify the extent of tissue removal. However, postoperative imaging is subject to variability related to timing, postoperative oedema, and difficulties in accurately distinguishing residual adenoma from the surgical cavity. Consequently, despite its growing role in research settings, imaging‐based assessment has not been uniformly adopted as a standard endpoint, reinforcing the variability in defining TURP completeness across studies.^21^

Importantly, the ability of a given surgical technique to remove prostatic tissue as completely as possible is influenced by multiple in vivo factors that extend beyond the intrinsic vaporization effect quantified in our experimental model. Bipolar TURP has consistently been associated with superior haemostasis and lower intraoperative bleeding compared with monopolar TURP, a characteristic that may indirectly impact the extent of tissue resection achieved in clinical practice. Reduced bleeding and a lower incidence of TUR syndrome, enabled by the use of isotonic saline irrigation, allow surgeons to prolong operative time and proceed more confidently toward the prostatic capsule without compromising patient safety.^15^, ^16^, ^20^ Consequently, differences observed in resected tissue weight between monopolar and bipolar techniques in vivo cannot be attributed solely to tissue vaporization, but rather to a complex interaction between energy modality, haemostatic efficiency, operative visibility, and surgical behaviour.

Beyond vaporization‐related tissue loss, the extent of tissue removal differs substantially between resection‐based techniques and enucleation due to intrinsic differences in surgical strategy. Enucleation is performed at the capsular plane, with controlled dissection and vascular interruption occurring at a limited and well‐defined interface, allowing more complete transition zone removal with less diffuse bleeding. Conversely, TURP relies on iterative tissue transection under continuous thermal exposure and bleeding, factors that inherently constrain both depth and completeness of resection.^9^, ^21^ As a result, differences in resected tissue weight across monopolar TURP, bipolar TURP and enucleation techniques cannot be interpreted as a simple function of vaporization alone, but rather as the combined effect of energy modality, haemostatic efficiency and the fundamental mechanics of each surgical approach.

The main limitation of this study lies in its in vitro design, which does not account for in vivo factors such as bleeding, capsular perforation, irrigation fluid temperature and prostatic tissue perfusion, all of which may influence tissue vaporization. Although the sample size was relatively small, it achieved adequate statistical power for the primary endpoint, which showed a narrow standard deviation, supporting the robustness of the observed differences between energy modalities.

## 5 CONCLUSION

The weight of adenomatous tissue removed during BPH surgery varies significantly by surgical technique. Resection‐based techniques cause 33% tissue loss due to vaporization and dehydration. MRI provides highly accurate adenoma volume estimates and should be considered a reliable tool for preoperative planning.

## AUTHOR CONTRIBUTIONS

Study concept and design: André B. Silva, Bruno R. Lebani and Fernando G. Almeida. Data acquisition: André B. Silva, Marcia E. Girotti and Hudson de Lima. Data analysis: André B. Silva and Bruno R. Lebani. Drafting of manuscript: André B. Silva, Eduardo R. Pinto, Luciano T. Silva and Denise S. Gouveia. Critical revision of the manuscript: André B. Silva, Milton Skaff and Fernando G. Almeida.

## CONFLICT OF INTEREST STATEMENT

None declared.
